# Minutes of the Pharmaceutical Meeting

**Published:** 1878-03

**Authors:** 


### MINUTES OF THE PHARMACEUTICAL MEETING.

PHILADELPHIA, February 19, 1878.

* * The following donations to the cabinet were received: From Mr. Neppach, a member of the present class, a specimen of *Barberry* or *Chittem Bark*, from Oregon, where it is used as a tonic and febrifuge; and from Mr. Joseph Jacobs, of Georgia, a specimen of the cotton plant (gossypium herbaceum) with root, stem and the cotton bolls in full development.

Mr. Ed. Gaillard read a paper upon formic acid, and in answer to a question, stated that he had not learned the particular purpose for which the physician wanted the acid. Prof. Maisch called attention to the spirit of ants, officinal in the German and other European pharmacopœias, which is merely an alcoholic solution of formic acid, but is still prepared from red ants.

Prof. Maisch stated that he was experimenting on bromide of iodine, a substance which has long been in use among photographers, in the daguerreotype process for rendering the plates more sensitive, but appears to be likewise used, to some extent, for medicinal purposes; he described it as exceedingly caustic, similar to bromine, and as evidently unfit for internal use in the concentrated state, or in large doses. Recently, through a typographical error in a medical journal, it had been directed instead of potassium bromide in a prescription for epilepsy; the error had been promptly corrected, but it seemed proper to direct attention to the caustic nature of the compound.

Mr. Charles L. Mitchell read a paper upon medicated bougies, giving their history and describing the various kinds in use, and the advantages of this method of medication; he exhibited specimens of the bougies as made by him, and also of Reynal's porte-remede, the former being more serviceable in appearance.

The chairman called the attention of the meeting to the collectsons of one hundred and seventy specimens of North American drugs and preparations made from some of them, which the College had directed to be sent to the Paris Exposition, and after the close of that exhibition to be presented to the Societe de Pharmacie of Paris. At the exhibition the collection will be in charge of Mr. Lindewald, a graduate of the College.

Attention was also called to the recent purchase made by the Board of Trustees of a large balance for scientific uses, especially valuable in enabling the members to have their weights and measures adjusted to the proper standard. An examination of the weights used in another city showed such discrepancies as to render it important that an examination of the weights used by the members of our profession should be instituted at once.

Mr. Shinn was called to the chair while Prof. Remington reported on a series of experiments undertaken by Mr. L. Wolff; having a considerable demand for the albumen of eggs, he was desirous of utilizing the yolks, and this led to the preparation of the fixed oil contained in the latter, and to an attempt of isolating the emulsifying principle, in which Mr. Wolff had been nearly successful. As obtained the principle will readily emulsify oil in water by simple agitation.

The use of fixed oils, in pharmacy, was brought forward, and among others benns oil, obtained from the seed of the sesamum orientale, was mentioned as a remarkably bland oil. Prof. Maisch stated that the chief objection to it was, that, though not a drying oil proper, it gradually became thick and ropy on exposure, and could, therefore, not be advantageously substituted for expressed oil of almonds; a sample of cold cream made by using this oil in place of oil of almonds was exhibited by Mr. Mitchell, and was considered equal to that made by the officinal formula excepting in color. Prof. Maisch said that it was quite a question how much of the commercial expressed oil of almonds was really such—great quantities of apricot and peach kernels were annually consumed to obtain oil from them. The test recommended by Hager will detect the substitution: equal parts of 25 per cent. nitric acid and the oil are agitated and warmed to about 120°F., when almond oil will furnish a white emulsion-like mixture, the other oils mentioned turning yellow or pinkish. (See also "*Amer. Jour. Phar.*," 1877, p. 595.)

A sample of so-called California rock soap had been shown at the November meeting; since that time it has been subjected to a chemical investigation by Mr. Betz, in the laboratory of the College, and proves to be a silicate of alumina with traces of calcium sulphate and iron, showing that it is a species of kaolin, and owes its detersive qualities to its mechanical rather than chemical action.

Prof. Maisch showed specimens of drugs that had been presented to him, as being very beautiful, carefully prepared, and of such superior quality as not often seen in the market; they were the root of archangelica officinalis, the flowering herb of erythræa centaurium, or European centaury; and mana in very large, white flakes.

There being no further business, on motion, the meeting adjourned.—*American Journal of Pharmacy.*

